# Feeble-mindedness

**Published:** 1915-04

**Authors:** 


### Feeble-mindedness.

A REVIEW BY THE EDITOR OF THE JOURNAL OF HEREDITY.

To the eugenist no problem is more immediate and serious than that of feeble-mindedness. Ever since eugenics became a recognized science its followers have devoted to feeble-mindedness what many have thought was an altogether disproportionate amount of time. With added knowledge the problem has increased rather than decreased in complexity and urgency. It is still far from being settled, but the past year has seen studies which do much to dispel the fog; and among these the most noteworthy by far is the work of Dr. H. H. Goddard, director of the research laboratory of the training school at Vineland, New Jersey, for feeble-minded boys and girls. As a result of five years of research, Dr. Goddard is able to present detailed pedigrees of 327 children in the institution, nearly all of them representing at least three generations. They are sufficiently complete, it appears, to furnish material from which anyone who cares to investigate can draw his own conclusions.

Feeble-mindedness has been defined as "a state of mental defect existing from birth or from an early age and due to incomplete or abnormal development in consequence of which the person affected is incapable of performing his duties as a member of society in the position of life to which he is born." It is, in other words, merely a state of arrested mental development (with some physical abnormalities in the lower grades), and is thus easily distinguished from insanity, which is a disordered rather than an arrested development of the mind. The feeble-minded are for convenience classed as idiots, imbeciles or morons, according to the point at which their development was arrested, the idiot being one whose mental age is two years or less, as measured by such a test as the Binet scale. An imbecile may have a development of from three to seven years, mentally, while the term "moron" in the United States designates one whose mental age is from eight to twelve years. As a fact, the moron class shades off imperceptibly into the normal bulk of society.

DEFECTIVES NUMEROUS.

The amount of feeble-mindedness in the community is much larger than anyone suspects who has not investigated conditions. In the United States Goddard thinks there are between 300,000 and 400,000 feeble-minded persons, but the distribution is very irregular; in some communities few are to be found, while in the State of New York alone the number has been placed as high as 30,000. These figures refer only to the feeble-minded who can actually be distinguished as such—the "latent" individuals. The number of "latent" individuals, those not actually feeble-minded themselves but carriers of the defect in their germ-plasm and capable of passing it on to their descendants, is necessarily vastly larger.

The taint, then, is so widespread that the student of heredity is amply justified in looking on it as the most important cacogenic factor in the community. Fortunately, however, not all feeble-mindedness is due to heredity. Just how much is due to other causes, no one can say. Goddard thinks that one-third of the cases examined in his work at Vineland may be ascribed to some other cause than inheritance. These appear to be due in some cases to a neuropathic ancestry, in others to accident before, at, or after birth, in others to some disease such as scarlet fever or spinal meningitis, during childhood. Cases of such origin are not transmitted to offspring, and, therefore, are of little importance to the genetist. By no means all cases where accidents are blamed for feeble-mindedness are really due to that cause, it appears, further investigation showing enough feeble-mindedness in the ancestry fully to account for the child's condition; on the other hand, the fact that a certain child is left feeble-minded by an attack of disease, while a dozen others who have it at the same time escape unscathed, indicates that here, too, a weakness of some sort in the family stock may explain the resulting arrest of mental development in the particular case. There are a number of supposed causes of feeble-mindedness which Goddard finds to have little reality, as far as his own experience goes. Thus there seems reason to doubt that parents' alcoholism arrests the child's mental development to the extent of leaving it among the feeble-minded. Paralysis, epilepsy, insanity or syphilis in the parents seem of themselves insufficient to account for a child's feeble-mindedness; neither can tuberculosis or consanguineous marriage explain such a result. Where these things occur they occur not as causes, but as corollaries or effects, of a defective germ-plasm.

THE "CRIMINAL TYPE."

On the other hand, feeble-mindedness itself is at the bottom of an amount of social abnormality which the ordinary sociologist—much less the layman—rarely realizes. The so-called "criminal type," Goddard believes, is merely a type of feeble-mindedness, a type misunderstood and mistreated, driven into criminality for which he is well fitted by nature. The chronic alcoholic is often to be explained by an arrest of mental development, rather than by original sin or moral perversity. The prostitute, in from one-fourth to two-thirds of the cases investigated in different cities, is found to be feeble-minded, and should be humanely segregated rather than punished or "reformed." The ne'er-do-wells of a community are usually found, if adequately tested, to be morons; the adult vagrant and the confirmed child truant usually belong to the same type. The expensive "special classes" of the public schools are filled with children a large part of whom are morons; an attempt is made to educate them, when an examination of their ancestry would show that it is humanly impossible to educate them in the way that their playmates are educated. In fact, such tests as those of Binet, wherever applied, have rarely failed to show that the number of social problems whose solution lies with genetics rather than with ordinary sociology is far greater than anyone except the eugenist realizes.

Unfortunately, the exact rôle of heredity in any given case can only be found by an investigation of the pedigree—a labor that involves much expense and time as well as the indispensable aid of a skilled field worker. Nay, the very presence of feeble-mindedness is often ignored, and an individual blamed for perversity, incompetence or stupidity, when the examination of a trained psychologist would show a discrepancy between mental age and physical age. The lower grades, the idiots and imbeciles, can indeed be distinguished by almost anyone, but the moron, particularly of the higher grade, is detected only by the careful observation of a competent investigator equipped with psychological tests and experience in using them. And even there one finds a border line where one cannot with confidence say whether the subject is normal or abnormal—these words being merely relative terms without exact definition.

"The feeble-minded in bulk—if we exclude special types of idiots—are not a special race, sharply differentiated from normal-minded folk," says Karl Pearson. "There is every grade of feeble-mindedness * * * and as far as mentality is concerned no sharp line can be drawn across the population, and those on one side of it treated as normal and those on the other as mentally defective,"—a statement of the case that I think Goddard and most other students would accept.

This fact of continuity has an important bearing on all work with the feeble-minded as a class. To the student of heredity it is particularly important, because most of the present-day studies of heredity start with the assumption that each character inherited is a unit. Can we speak of a unit character, when it shades off imperceptibly into another—can we call feeble-mindedness a unit, when no line can be drawn between it and its supposed alternative or "allelomorph," a normal mind?

THE UNIT CHARACTER QUESTION.

This question has caused bitter disagreement among eugenists. The earlier students of the subject assumed that it was a unit character and interpreted their pedigrees in that light. They called it a recessive trait, normal mentality being dominant. From the very beginning psychologists had on the whole refused to assent to this proposition. Goddard himself "confesses to being one of those psychologists who find it hard to accept the idea that the intelligence even *acts like a unit character,"* although his own figures force him to say that "there seems to be no way to escape such a conclusion." But the question was not really made a crucial one among genetists until the publication of three bulletins from the Galton Laboratory of London, devoted largely to an attack on exactly this feature of recent American work in eugenics.

In the first one Dr. Heron, the assistant director, devoted himself wholly to a destructive criticism of studies which considered mental defect from a Mendelian viewpoint—which assumed, in short, that feeble-mindedness was a unit character and so behaved in inheritance. He was answered by some of the men he attacked; but as the controversy was fairly well aired in the daily as well as the scientific press, it will not be renewed here.

Following this came another contribution from London, in which measurements of the intelligence of school children in Stockholm were presented to prove that feeble-mindedness was not a unit character but showed continuity with the normal population. Still later Karl Pearson published in the same series a lecture reviewing the whole situation, and pointing out the need for some accurate measurement of the higher grades of mental deficiency. "That a real measure will be found—short of the experimental method of testing actual success or failure in the rough and tumble of life—I am convinced," he concludes, "but I doubt whether it has been found at present and its discovery will not be expedited by any scientific dogma that asserts all mental defect is of one kind, and is due to the absence of a determiner, a lack which the feeble-minded share with our ape-like ancestors."

The smoke of controversial battle must not obscure from the public the fact that as to the hereditary nature of much if not most feeble-mindedness there is no doubt. This alone would suffice to justify a eugenic campaign. The further question of how it is inherited is purely a technical one. Nevertheless, it is one which has great importance to society as well as to the professional genetist. What, then, is the layman to think about the way in which this condition is transmitted?

It seems impossible to overlook the ease with which the puzzle can be explained by the hypothesis that feeble-mindedness is due to not one but many unit characters, which in general, *but not always,* cling together in a group when transmitted. When they stick together in one group they produce the appearance of a single unit character, and thus yield the approximate Mendelian proportions which Goddard found when he tabulated his matings, and which his predecessors also found in their researches. But when some other factor comes into play this group may be broken up and only a part of the units passed on to a given individual. They may be enough in number to produce obvious feeble-mindedness; they may be so few that the individual who receives them appears, in any ordinary environment, to be little inferior to his comrades.

### NEED FOR MORE RESEARCH.

If this hypothesis be the true explanation of the behavior of feeble-mindedness in heredity the antagonism between the biometrists with their insistence on continuity and the Mendelians, with their insistence on the unit character, is only apparent. Neither side at present accepts such a solution, but it seems probable that the further prosecution of genetic studies will result in a more general acceptance of the idea that supposed unit characters are multiple and that visible traits are complex in nature.

But as I have already said, we must not get so much interested in a question of secondary importance as to forget the point of first importance—namely, that feeble-mindedness is a widespread defect, largely due to heredity, which threatens to lower the intellectual level of the whole race unless careful selection in mating keeps it from infecting more sound stock each year. As feeble-mindedness involves a lack of self-control and an inability to understand ethical questions, the possessor of it cannot be expected voluntarily to take any steps which will prevent the transmission of his defect. With society is the responsibility for protecting itself.

But the opponents of eugenics object. Nature will take care of the whole matter. These diseased conditions of the germ-plasm "run out"; the stream always tends to purify itself. "A study of the charts here presented," Dr. Goddard remarks, "will hardly be found reassuring in this direction." In the absence of any interference the number of feeble-minded usually becomes larger with each generation; if they are of the very lowest grade it is true that they leave no descendants, but among the morons the taint is more likely to spread than not. And even in cases where it seems to have died out, where no feeble-mindedness appears for three or four generations, we cannot be sure that the condition has not become merely latent. The genetist, indeed, who has seen exactly analogous cases in his breeding experiments, will feel quite sure that it is merely latent, ready to appear again when the proper mating is made. There is no safeguard for society in a dependence on some mystical help from nature; it must protect itself by deliberate intervention.

At present society fulfills this obligation in a very inadequate manner by segregating, in some states, a small part of the most hopeless grades of defectives. Even then they are not always prevented from propagating their kind. There is general agreement that effective segregation should be extended, but it is, of course, difficult to say who should and who should not be included. Pearson thinks that it is merely "a matter of practical utility where we draw the line which shall legally define mental defect for purposes of segregation. It certainly should not be done under four years mental defect judged by adequate Binet tests. This will only cut off about 20 to 30 per cent of those at present classed as feeble-minded in the special schools. The remaining 70 or 80 per cent may be, and probably are, incapable of fending for themselves in ordinary life; they are also socially inefficient, but ultimately, on other grounds, temperamental or moral, not merely intellectual; they take a view of life which is in distorted perspective, and they are out of harmony with their economic or social surroundings. They may be more dangerous than those in whom true *mental* defect is far greater, and they may more urgently stand in need of segregation," because while the groveling idiot is unlikely to become a parent, the moron is almost certain to do so, either legitimately or illegitimately, unless prevented by society from doing so.

How shall this restraint be exercised? In the first place, says Dr. Goddard, as many of the high grade defectives as possible must be effectively "segregated" in their own homes, by the intelligent action of their own relatives. As to the rest, the lower grades must obviously be gathered together in colonies, not only for the protection of society, but for their own protection. But if the higher grades are to be included, it will mean the establishment of at least a thousand colonies, each containing 300 or 400 individuals—a burden which the nation is not likely to assume at present.

"The facts," to Dr. Goddard, "show that we must colonize as many of the feeble-minded as we possibly can, that we must sterilize some, and then we discover that we have only tithed the problem, we have not solved it." The rest—all of them, of course, representing the higher grades—should, he says, be educated.

Impossible?

"That depends on our definition of education," he answers. They can be trained, if recognized and taken in hand at an early enough age, to do many kinds of work which do not demand the possession of judgment or real intelligence, but which depend rather on habit. Few of them can be taught usefully to read, write, or count, but there are many kinds of manual labor that they can be trained to do with sufficient proficiency to pay for their cost of maintenance. Even should the community find itself unable to stop altogether the production of this class, at present, "may it not be possible that we will find use for all these people of moderate intelligence, and that the production of so many high grade feeble-minded is only the production of so many more people who are able and willing to do much of the drudgery of the world, which other people will not do?"

There remains a question of first importance to the eugenist—the relation to marriage selection of the moron who is left at liberty. Here students sometimes seem to fall into no more than two classes: those who propose wholesale sterilization and those who propose nothing. Dr. Goddard takes a middle course, thinking sterilization adequate to dispose of "a narrow zone" of cases; for the rest, it appears that general education of the public to realize that morons exist in large numbers, in all classes of society, and that they cannot by outward appearance be detected, will do much, perhaps all that is possible, to abolish the problem. When the man or woman contemplating marriage realizes the desirability of investigating the ancestry of a prospective mate, the infection of sound stocks by marriage between normal persons and high grade morons or those who, wholly normal in themselves, are carriers of latent feeble-mindedness, will largely cease.—*Journal of Heredity.*

---

HIS DEFECT.—"Dr. Jibs seems to be looked down on by the rest of the profession."

"That's on account of his unprofessional conduct."

"In what way?"

"Why, the man will use any treatment at all which will cure the patient!"—*Baltimore American.*

---

A YOUNG DOCTOR received late one evening a note from three of his fellow practitioners: "Please come over to the club and join us in a game of poker." "Emilie, dear," he said to his wife, "here I am called away again.' It is an important case—there are three other doctors on the spot already."—*Riesel Rustler.*

---

A UNIQUE little brochure (24 pages) on "Clinical Symptomatology" has already been distributed to the medical profession by the Purdue Frederick Company, who prepare the well-known Gray's Glycerine Tonic Comp. This consists of a number of tables or charts giving the common or usual "symptom-complex" of each of sixty different diseases and will prove of exceptional value for reference purposes. If any physician did not receive a copy of "Clinical Symptomatology" he can easily obtain a copy by addressing The Purdue Frederick Company, 135 Christopher Street, New York City.

